# Chemoresistance of Human Monocyte-Derived Dendritic Cells Is Regulated by IL-17A

**DOI:** 10.1371/journal.pone.0056865

**Published:** 2013-02-18

**Authors:** Selma Olsson Åkefeldt, Carine Maisse, Alexandre Belot, Marlène Mazzorana, Giulia Salvatore, Nathalie Bissay, Pierre Jurdic, Maurizio Aricò, Chantal Rabourdin-Combe, Jan-Inge Henter, Christine Delprat

**Affiliations:** 1 Childhood Cancer Research Unit, Department of Women's and Children's Health, Karolinska Institutet, Karolinska University Hospital Solna, Stockholm, Sweden; 2 CNRS, UMR5239, Laboratoire de Biologie Moléculaire de la Cellule, Lyon, France; 3 Ecole Normale Supérieure de Lyon, Lyon, France; 4 Université de Lyon, Lyon, France; 5 Université de Lyon 1, Villeurbanne, France; 6 UMS3444/US8, Lyon, France; 7 Hospices Civils de Lyon, Hôpital Femme Mère Enfant, Bron, France; 8 CNRS, UMR5242, Institut de Génomique Fonctionnelle de Lyon, Lyon, France; 9 Università degli Studi di Firenze, Firenze, Italy; 10 Department Pediatric Hematology Oncology, Azienda Ospedaliero-Universitaria Meyer Children Hospital, Florence, Italy; 11 INSERM, U851, Lyon, France; 12 Institut Universitaire de France, Paris, France; Leiden University Medical Center, The Netherlands

## Abstract

Dendritic cells initiate adaptive immune responses, leading either to control cancer by effector T cells or to exacerbate cancer by regulatory T cells that inhibit IFN-γ-mediated Th1-type response. Dendritic cells can also induce Th17-type immunity, mediated by IL-17A. However, the controversial role of this cytokine in cancer requires further investigations. We generated dendritic cells from peripheral blood monocytes to investigate lifespan, phenotype and chemoresistance of dendritic cells, treated with IL-17A with or without IFN-γ. Studying the expression of Bcl-2 family members, we demonstrated that dendritic cells constitutively express one pro-survival Bcl-2 member: MCL1. Immature dendritic cells were CD40^low^HLADR^low^ CD1a^+^ MCL1^+^, did not express CD14, CD68 or BCL2A1, and displayed a short 2-day lifespan. IL-17A-treated DC exhibited a semi-mature (CD40^high^ HLADR^low^) pre-M2 (CCL22^+^ CD206^+^ CD163^+^ IL1RN^+^ IL-10^−^ CXCL10^−^ IL-12^−^) mixed (CD1a^+^ CD14+ CD68^+^) macrophage-dendritic cell phenotype. They efficiently exerted mannose receptor-mediated endocytosis and did not produce superoxide anions, in the absence of TLR engagement. Interestingly, IL-17A promoted a long-term survival of dendritic cells, beyond 12 days, that correlated to *BCL2A1* induction, a pro-survival Bcl-2 family member. *BCL2A1* transcription was activated by NF-κB, downstream of IL-17A transduction. Thus, immature dendritic cells only express MCL1, whereas IL-17A-treated dendritic cells concomitantly expressed two pro-survival Bcl-2 family members: MCL1 and BCL2A1. These latter developed chemoresistance to 11 of the 17 chemotherapy agents tested. However, high doses of either vinblastine or cytarabine decreased MCL1 expression and induced dendritic cell death. When IL-17A is produced *in vivo*, administration of anti-IL-17A biotherapy may impair dendritic cell survival by targeting BCL2A1 expression. Consequently, depending on the effector or regulatory role of dendritic cells, blocking IL-17A, may be either dangerous or beneficial for cancer outcomes, thus contributing to the apparent controversy around the role of IL-17A in cancer.

## Introduction

About 15% of human cancers are associated with inflammation [1]. A topical question is whether it can be advantageous to add biotherapy to conventional chemotherapy for affected patients. Crohn's disease and Sjogren's syndrome are IL-17A-dependent chronic inflammatory diseases that are associated with an increased risk of colon cancer [2] and lymphoma[3] , respectively. IL-17A is a proinflammatory cytokine associated with several chronic inflammatory diseases [4], which regulates the activities of NF-κB transcription factor and mitogen-activated protein kinases to stimulate the expression of IL-6, cyclooxygenase-2 and nitric oxide. Its receptor is composed of IL-17RA and IL-17RC. IL-17RA is ubiquitously expressed, with particularly high levels in immune cells, while IL-17RC is preferentially expressed in non-immune cells [5]. It is not clear how myeloid cells, which are IL-17RA**^+^**/IL-17RC**^−^,** bind IL-17A. Recent crystallographic and computational analysis have suggested that IL-17RA is a common chain shared by all receptors [6], and the exact contribution of IL-17RC is under investigations.

The role of IL-17A in cancer remains controversial with protumor (pro-angiogenic) versus antitumor (immune) effects. In mouse models of fibrosarcoma or colon adenocarcinoma, over-expression of IL-17A by cancer cells increased tumor growth [7]. This effect was indirectly mediated by a VEGF-dependent pro-angiogenic activity. By contrast, in hematopoietic tumors such as mastocytoma or plasmacytoma, IL-17A prevented tumor development by increasing the generation of tumor specific cytotoxic T lymphocytes [8]. This effect is probably also indirect since activation of naïve CD8^+^ T lymphocytes usually relies on IFN-γ-mediated Th1-type response and primarily requires dendritic cells (DC), presenting tumor peptides on their MHC molecules. The role of IL-17A on DC is therefore a challenge to address.

Since more than twenty years, immunologists have studied the biology of monocyte-derived DC, obtained with GM-CSF and IL-4, *in vitro*. The *in vivo* relevance was finally established in the mouse in 2010 [9]. We have reported that IL-17A robustly activates survival pathways in monocyte-derived DC, but not monocytes, *in vitro* [10]. However, the IL-17A-dependent mechanism that controls DC survival has not been established.

Proteins of the B-cell lymphoma 2 (Bcl-2) family regulate survival and sensitivity to apoptosis by governing mitochondrial outer membrane permeabilization and release of cytochrome c from mitochondria in the intrinsic apoptotic pathway [11]. The main function of the pro-survival Bcl-2 proteins is to counteract the activation of the pro-apoptotic Bcl-2 proteins which include the BH3-only proteins, BAK and BAX. Once activated, BH3-only proteins activate BAX or BAK that form pore in the outer mitochondrial membrane, mediating cytochrome c release. Thus, pro-survival members can either directly inhibit BAX, BAK or sequester and inactivate BH3-only proteins in the cytoplasm. Myeloid cell leukemia sequence 1 (*MCL1)* was first discovered as a pro-survival member in a human myeloblastic leukemia cell line [12]. Bcl-2-related protein A1 (*BCL2A1)*, discovered in 1995 in B cell lymphoma, is another pro-survival member preferentially expressed in lymphoid cells [13], [14]. Due to their central function in the apoptotic machinery, Bcl-2 proteins are often deregulated in the sense of a pro-survival effect, in cancer. Small molecules that inhibit pro-survival Bcl-2 proteins in cancer cells counteract chemoresistance and cure cancer in a high percentage of mice [15].

In this study, we reveal that the long-term survival mechanism induced by IL-17A in human DC is under the control of *BCL2A1* induction. In addition, we studied the phenotype and chemoresistance of IL-17A-treated DC to 17 drugs, *in vitro*, and provide new insights on combining cytotoxic compounds with anti-IL-17A biotherapy.

## Materials and Methods

### Healthy donors

We obtained blood samples from healthy adult volunteers at the Etablissement Français du Sang (Lyon, France). The local ethics committee (Research Committee for the Hospices Civils de Lyon) approved this study and we obtained written informed consent from each subject (national procedure used for blood donations).

### Reagents

We purchased recombinant human GM-CSF, IFN-γ, IL-4 and IL-17A from PeproTech (Neuilly-sur-Seine, France). Flow cytometry: CD14, CD68, CD1a, HLA-DR, CD40, CD206, CD163 and isotype controls were purchased from Becton Dickinson (Le Pont de Claix, France), anti-BCL2A1 (3401 anti-A1) from BioVision (San Francisco, USA), anti-MCL1 (Y37) from Abcam (Cambridge, UK) and Bay-11-7085 (2 µM), inhibitor of the classical NF-κB pathway, from Calbiochem (Merck, Darmstadt, Germany). Toxic compounds: dexamethasone, 6-mercaptopurine and fludarabine were purchased from Sigma Aldrich (St Louis, MO, USA) and the remaining drugs were kindly provided by the Karolinska University Hospital pharmacy (see Methods S1).

### Cultures

CD14^+^ monocytes were purified (>95% CD14^+^) from the peripheral blood by ficoll and percoll gradients, followed by negative magnetic depletion of cells expressing CD3 or CD56 or CD19. Monocytes were treated 6 days with 50 ng/ml GM-CSF and 500 U/ml IL-4 in RPMI (Life Technologies, Carlsbad, CA, USA) supplemented with 10% FCS, 10mM Hepes, 2 mM L-glutamine, 40 µg/mL gentamicin (Life Technologies) [16]. Cytokines were then removed by washing DC twice in cytokine-free medium. Flow cytometry analysis was routinely used for quality control of the immature DC phenotype CD14^−^CD1a^+^MHC-II^+^CD83^−^(>98%), *in vitro*. The very day, DC at day 0, were seeded at 4,800 cells/mm^2^ in the presence or not (None) of IL-17A with or without IFN-γ. Cytokines were added at 2 ng/mL, otherwise indicated on the figure, and replenished every week. Toxic compounds were added either concomitantly with IL-17A and IFN-γ or 24 hours later, as indicated.

### DC survival and fusion efficiency quantification by TRAP and Hoechst staining

In long-term cultures, cells became adherent and underwent cell fusion. As previously described [10], [16], we analyzed survival and cell fusion after tartrate resistant acidic phosphatase (TRAP) and Hoechst double staining. We used the Leukocyte acid phosphatase kit (Sigma-Aldrich) to visualize the cytoplasm stained by the pink product resulting from TRAP activity. Then we stained the nuclei with 10 µg/ml of Hoechst 33342 (Sigma), a blue fluorescent DNA dye, for 30 min at 37°C. After two washes and fixation with 1% formaldehyde, we counted (N) the total number of active nuclei in viable mono or multi-nucleated cells, per well, over time. 10^6^ DC per well were put in culture, at day 0. Survival percentage was calculated: [(N)/10^6^×100]. We counted (n) the total number of nuclei included in MGC, per well. Cells were considered as MGC when containing strictly more than 2 nuclei. Then we calculated the fusion efficiency as the percentage of the total nuclei, included in MGC: [(n)/(N)×100].

### CFSE and CD14-PE labeling for proliferation study

DC were suspended at 10^7^ cells/mL in α-MEM containing 2% FCS. After 15 minutes of incubation in the presence of 10 µM carboxyfluorescein diacetate, succinimidyl ester (CFSE), the CFSE incorporation was blocked by the addition of a large excess of α -MEM, containing 2% FCS [16]. DC were then washed twice by centrifugation at 1500 rpm for 10 minutes at 4°C in α -MEM containing 2% FCS and seeded in α -MEM containing 10% FCS with indicated cytokines. Cells were then harvested at day 7 by a trypsin treatment (Sigma-Aldrich) and scraping, and finally immunostained with a CD14-PE antibody. The expression of CD14-PE and CFSE was quantified on an LSRII (Becton Dickinson) and analyzed using FlowJo software.

### DiOC_6_ and PI labeling for cell survival quantification by flow cytometry

From day 0 to 7, >95% of the IL-17A-treated DC were mononucleated. Cell survival was analyzed by flow cytometry after DiOC_6_(3) (3,39-diexyloxacarbocyanine) and propidium iodide (PI) double staining. Cells were incubated 15 min at 37°C with 40 nM DiOC_6_ (Molecular Probes) in culture medium to evaluate mitochondrial transmembrane potential (Δψ*m*). Viable cells have stable Δψ*m* whereas Δψ*m* decreases with cell commitment to apoptosis. PI (0.5 µg/ml) was added before flow cytometry analysis of the cells and incorporated into DNA of dead cells whose membrane is permeabilized. Apoptotic cells are DiOC_6_^−^PI^+^, while living cells are DiOC_6_^+^PI^−^. 10^6^ DC/well (survival>98%) were seeded at 4,800 cells/mm^2^, at day 0. The total number of viable cells per well was quantified by a time-monitored flow cytometry analysis during 2 min at high speed (1 µl/s). Cell survival was calculated as the percentage of viable cells at day 7 related to day 0, for 10^6^ DC introduced at day 0. In absence of cell division, cell death percentage is the complement of the survival percentage to 100.

### Flow cytometry staining

Immunostaining of cells was performed in 1% BSA and 3% human serum-PBS. We used 2 µg/ml of primary or secondary PE-F(ab′)_2_ goat to mouse IgG, 115-086-062, Jackson Immunoresearch (West Grove, PA, USA) antibodies. For intracytoplasmic staining, we blocked the Golgi apparatus with BD GolgiStop™, fixed and permeabilized the cells with the Cytofix/Cytoperm reagents according to procedures from the manufacturer (Becton Dickinson). Fluorescence was quantified on a LSRII (Becton Dickinson) and analyzed using FlowJo software.

### Affymetrix genechip study

RNA was purified from DC, either untreated, or cultured for 12 days with indicated cytokines: after cell lysis, extraction in Trizol (Invitrogen, Saint Aubin, France) and purification on MEGAclear column Ambion (Invitrogen) to reach an RNA integrity number >9 with Agilent bioanalyser, “ProfileXpert” (www.profilexpert.fr) performed the chip study (see Methods S1).

### Functional analysis of MR-mediated endocytosis

The endocytotic capacity of cells was analyzed with dextran FITC, a probe for MR-mediated endocytosis. As, in addition to receptor-mediated endocytosis, there is some uptake of dextran FITC by pinocytosis, preincubation with mannan was performed to block the MR-mediated endocytosis and delineate MR-mediated endocytosis from the background fluorescence of dextran FITC uptake by pinocytosis. Cells were cooled down in an ice water bath. Time course studies were performed at 0, 10 and 30 min by re-warming the cells in a water bath to 37°C in the presence of dextran FITC (1 mg/ml), or a 10 min pre-incubation with mannan (1 mg/ml) followed by dextran FITC (1 mg/ml). For quantitative evaluation of the receptor density, two-dimensional dot plot analysis was performed for the respective monocytes, derived DC, and these DC treated for 7 days with IL-17A. Populations were gated out manually. The mean fluorescence intensity (MFI) of the FITC signal was determined and fluorescent dye uptake was quantified by calculating the fluorescent index (FI) for the different time points: FI = [MFI (10, 30 min)−MFI (0 min)]/MFI (0 min). This normalization procedure makes the entire procedure resistant against donor variability.

### Real-time quantitative PCR

Total RNA from 2 millions of cells was extracted using Trizol® (Invitrogen) and RNeasy Mini Kit® (Qiagen, Düsseldorf, Germany) to reach an RNA integrity number >9 with Agilent bioanalyzer. RT-PCR reactions were performed with SuperScript® II Reverse Transcriptase (Invitrogen). One µg total RNA was reverse-transcribed using oligo(dT)12-18 Primers (Invitrogen). For expression studies, 25 ng of cDNA were amplified in Stratagene Mx3000P apparatus (Agilent Technologies), using the QuantiTect® SYBR®Green PCR Kit (QIAGEN). Primer sequences were as follows: BCL2A1, ACA GGC TGG CTC AGG ACT ATCT (forward), CTC TGG ACG TTT TGC TTG GAC (reverse); GAPDH, CAC CCA CTC CTC CAC CTT TGAC (forward), GTC CAC CAC CCT GTT GCT GTAG (reverse); TBP, QuantiTect® primers specific Hs_TBP_1_SG QuantiTect Primer Assay (Qiagen). All samples were normalized to expression of GAPDH or TBP.

### Western blot analysis

Three millions cells were harvested, sonicated and lysed 1 h at 4°C with RIPA buffer containing protease inhibitor cocktail (Roche, Indianapolis, USA). Cellular debris were pelleted by centrifugation (10,000 g 15 min at 4°C) and protein extracts (100 µg per lane) were loaded onto a 12% SDS-polyacrylamide gel and blotted onto PVDF sheet (Bio-Rad Laboratories, Hercules, CA, USA). Filters were blocked with 5% BSA in PBS/0.1% Tween 20 (PBS-T) for 2 h and then incubated over-night at 4°C with anti-BCL2A1/BFL1, 0.9 µg/mL in PBS-T (rabbit polyclonal ab75887, Abcam, Cambridge, UK). After three washes with PBS-T, filters were incubated 1 h with Biotin-conjugated goat anti-rabbit IgG, 2 µg/mL in PBS-T, 5% BSA (Molecular Probes/Invitrogen, Eugene, Oregon, USA). After three washes with PBS-T, filters were incubated 1 h with HRP-conjugated Streptavidin (StrepTactin-HRP, Bio-Rad Laboratories, Hercules, CA, USA) dilution 1∶50,000 in PBS-T, 5% BSA. Detection was performed using Immun-StarTM WesternCTM Kit chemiluminescence system (Bio-Rad Laboratories, Hercules, CA, USA). Actin staining was realized using a rabbit polyclonal anti-βActin from Santa Cruz (sc-130656, Santa Cruz, CA, USA).

### Immunocytofluorescence labeling of p65/RelA

4×10^5^ monocyte-derived dendritic cells were cultured in 8-well Lab-Tek™ Chamber Slide™ System (Nunc, Thermo Scientific), eventually with IL-17A (2 ng/mL). At the indicated times, the chamber slides were gently centrifugated and cells were fixed with PBS containing 4% paraformaldehyde for 30 min at 4°C. Cells were permeabilized with PBS containing 0.2% Triton X-100 for 20 minutes at room temperature. After saturation (30 minutes at room temperature in PBS 1% BSA, 3% human serum), cells were incubated 2 h with 4 µg/mL p65/anti-RelA in PBS 1% BSA (C-20, Santa Cruz Biotechnology, California, USA). After 3 washes in PBS 1% BSA, cells were incubated 30 minutes at room temperature in the dark with Alexa Fluor 647 Goat anti-rabbit IgG (10 µg/mL, Molecular Probes, Invitrogen, Carlsbad, California USA). After 3 washes, cells were mounted in Dako Fluorescent Mounting medium (Dako, Denmark), and immunostaining images were analyzed using a Leica TCS-SP5 laser scanning confocal microscope (Leica, Wetzlar, Germany).

### Statistical analysis

Polynomial statistical analysis and Mann-Whitney U test from GraphPad Prism 5 software were applied to detect differences between subgroups; the cutoff level of p<0.05 was regarded significant.

## Results

### IL-17A promotes long-term survival of a new non-proliferating myeloid cell subset with a pre-M2 macrophage-DC phenotype

**Figure 1:**
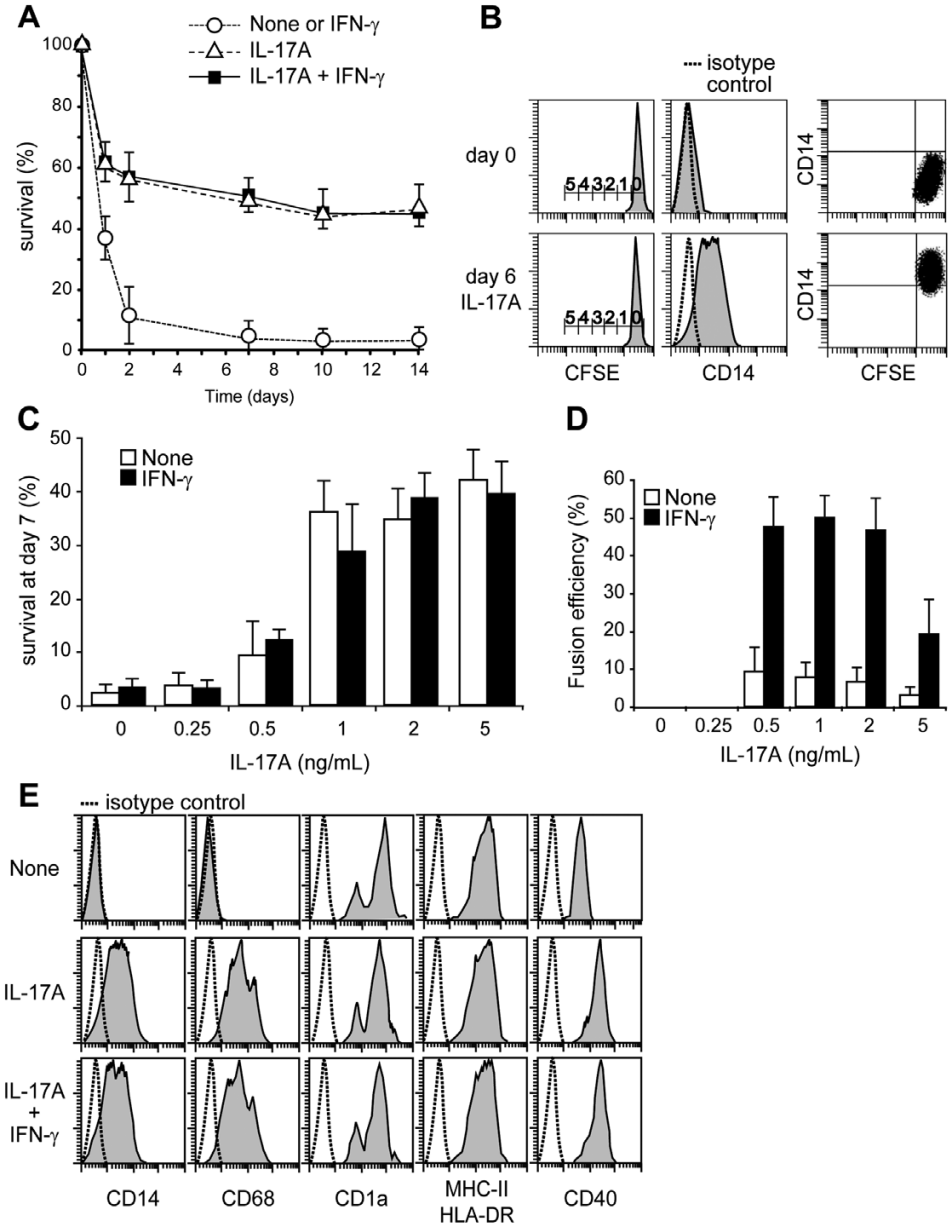
Survival, proliferation, fusion and phenotype of IL-17A and IFN-γ-treated DC. (**A**) Kinetic study of cell survival in long-term culture of DC with indicated cytokines, assessed by TRAP and Hoechst staining. Mean and SD of n = 7. (**B**) Flow cytometry analysis of CD14 expression and DC divisions (CFSE staining) at day 0 and 6 after addition of IL-17A. Scale bar indicates number of cell divisions, representative of n = 3. (**C**) IL-17A-dependent dose response study of DC survival, at day 7 of culture with indicated cytokines, assessed by DiOC_6_ and PI staining. Mean and SD, n = 3. (**D**) IL-17A-dependent dose response study of DC fusion, at day 12 of culture with indicated cytokines, assessed by TRAP and Hoechst staining. Mean and SD, n = 4. (**E**) Expression (gray) of CD14, CD68, CD1a, HLADR and CD40 by monocyte-derived DC at day 0 (None) and 48 h after culture with IL-17A +/− IFN-γ, representative of n >5.

**Figure 2:**
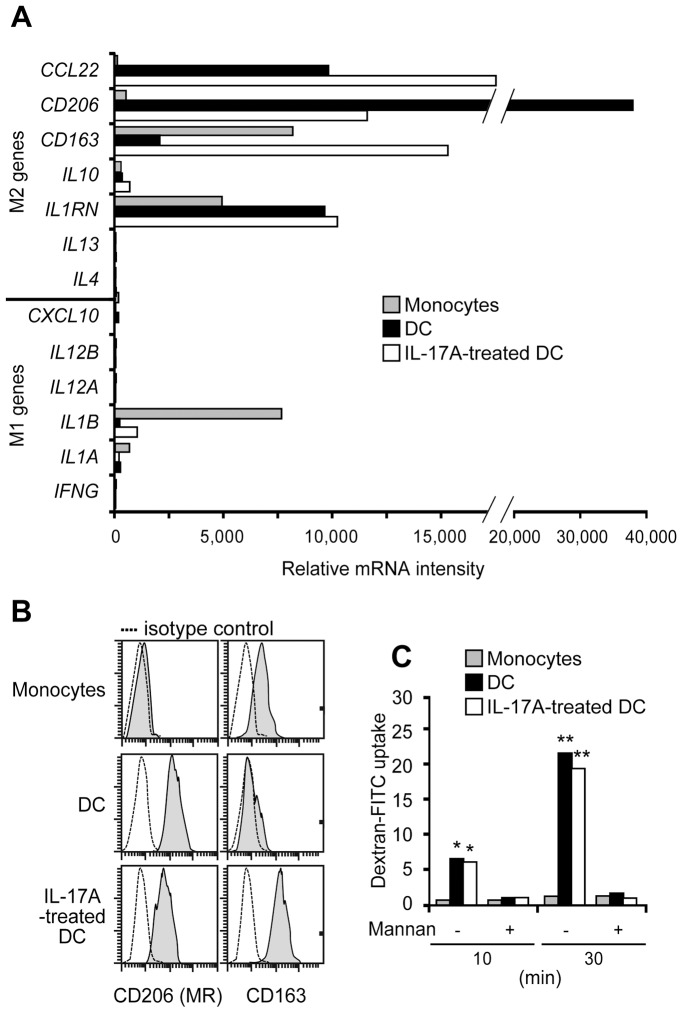
Comparative phenotypic analysis of monocytes, DC and IL-17A-treated DC. Comparative study of monocytes (freshly isolated), immature DC (derived from monocytes) and IL-17A-treated DC for (**A**) mRNA intensities of M1 versus M2 macrophage-related genes by microarray, representative of n = 4; (**B**) expression (gray) of CD206 (or mannose receptor, MR) and CD163, representative of n = 3; (**C**) uptake of dextran FITC (MR-mediated endocytosis), in the presence of mannan competitor when indicated, mean FI values of n = 3, SD <10% not shown. Statistical significance was determined by the Mann-Whitney test, *, significant p<0.05; **, very significant p<0.01. IL-17A treatment administrated for (**A**) 12 days and (**B,C**) 48 hours.

DC lifespan controls the priming of adaptive immune responses. *In vitro*-generated DC, from human monocytes cultured with GM-CSF and IL-4, cannot survive more than 48 h in cytokine-free medium or with IFN-γ alone (**Fig. 1A**). Notably, IL-17A treatment rescued around half of DC from death, and these cells then survived long-term in culture, without GM-CSF and IL-4, if IL-17A was replenished once a week. In the presence of IL-17A, no DC proliferation was observed (**Fig. 1B**). IL-17A dose-dependently induced DC survival (**Fig. 1C**). IFN-γ had no effect on IL-17A-induced survival, whereas it potentiated IL-17A-induced DC fusion quantified at day 12 (**Fig. 1D**). In the presence of GM-CSF and IL-4, CD14^+^CD1a^−^ human monocytes differentiated into CD14^−^CD68^−^CD1a^+^HLA-DR^+^CD40^low^ immature DC (**Fig. 1E**). IL-17A-treated DC still expressed the CD1a DC marker at 48 h, but also acquired macrophage (CD14/CD68) markers. The intensity of HLA-DR remained unchanged, while the CD40 expression was upregulated by IL-17A treatment. Addition of IFN-γ did not affect the IL-17A-mediated semi-mature mixed DC-macrophage phenotype. The up-regulation of CSF1 by IL-17A reinforced the CSF1/CSF1R axis in the DC (**Fig. S1**). Therefore the phenotype of IL-17A-treated DC may be closer to a macrophage rather than a DC. We studied whether these myeloid cells were polarized towards M1 or M2 type macrophages (**Fig. 2**). We studied the M1 and M2 genes in monocytes, DC and IL-17A-treated DC. Although the IL-17A-treated DC did not exhibit all the characteristics of M1 or M2 macrophages, their phenotype looked like a pre-M2 phenotype with high expressions of CCL22, CD206, CD163 and IL1RN (IL-1 receptor antagonist) mRNAs, while the M1 genes were weakly or not expressed (**Fig. 2A**). Flow cytometry confirmed high CD206 (mannose receptor) and CD163 protein expressions in IL-17A-treated DC (**Fig. 2B**). As assessed by the uptake of dextran-FITC, CD206-mediated endocytosis was functional in IL-17A-treated DC (**Fig. 2C**). In the absence of Toll receptor engagement, we noticed neither an activated M2 microenvironment, since IL-4, IL-13 and IL-10 were not detected (in the transcriptome and by ELISA), nor an activated M1 microenvironment, since IFN-γ, IL-1β, IL-12 and superoxide anion production (nitro blue tetrazolium assay) were also below the technical background (data not shown). Altogether, these data demonstrate that IL-17A strongly modifies the human DC phenotype into CD1a^+^CD14^+^CD68^+^ long-term surviving mono and multinucleated myeloid cells, displaying a pre-M2 non-activated phenotype.

### IL-17A induced BCL2A1 transcription in human DC

**Figure 3:**
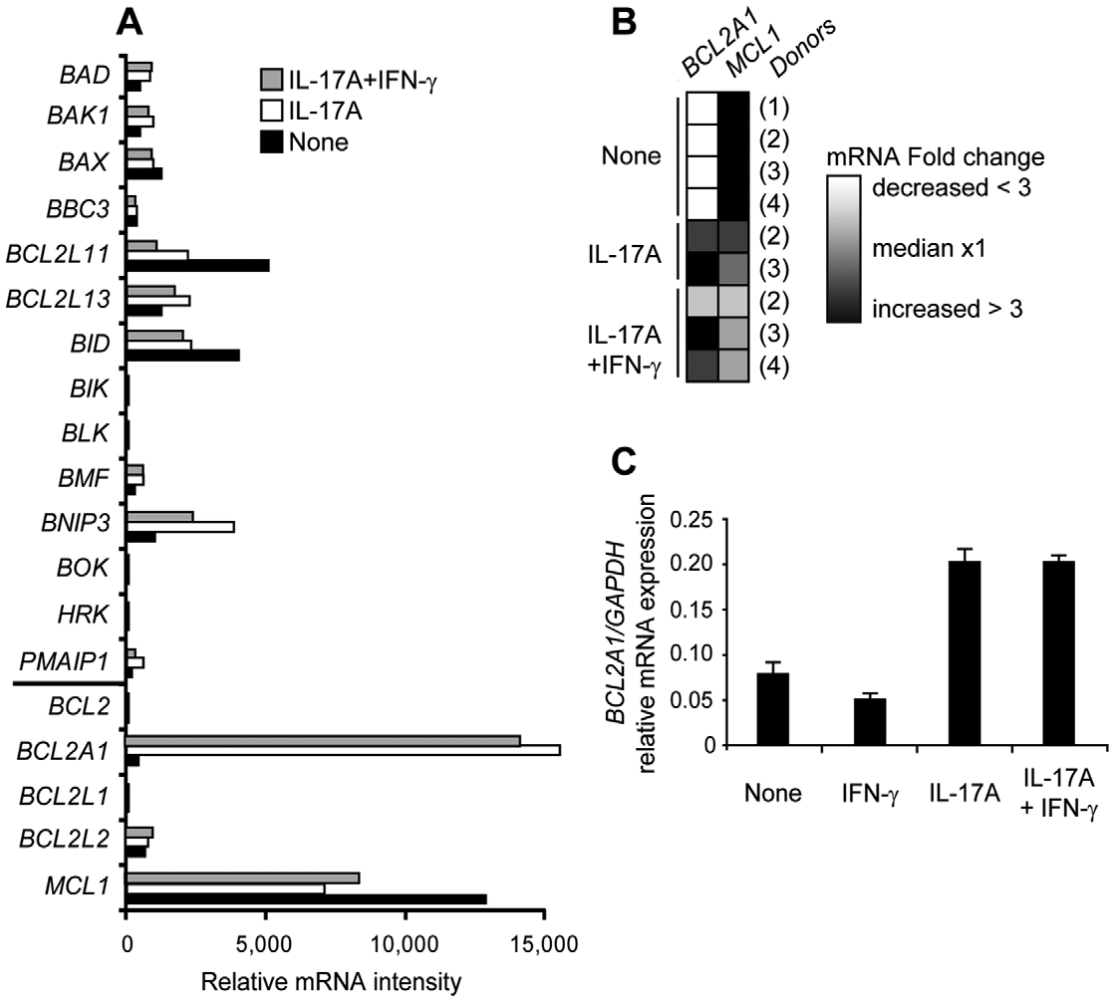
Bcl-2 family mRNA expression in IL-17A and IFN-γ-treated monocyte-derived DC. (**A,B**) DC at day 0 (None) or treated 12 days with IL-17A +/− IFN-γ. (**A**) Whole study of *Bcl-2* family mRNA intensities by microarray, left horizontal bar on Y axis separates pro-apoptotic members (top) from pro-survival members (bottom), representative of n = 4. (**B**) Fold change heat map of *BCL2A1* and *MCL1* mRNA from normalized experiments and four donors (1) to (4), as indicated. White and black color gradients reflect decrease and increase in fold change, respectively. (**C**) mRNA levels of *BCL2A1* measured by real-time PCR in DC at day 0 (None) and 48 h of culture with IFN-γ or IL-17A or both. Mean and SD of duplicate of relative gene expression (compared with GAPDH) for one donor, representative of *n = *3.

**Figure 4:**
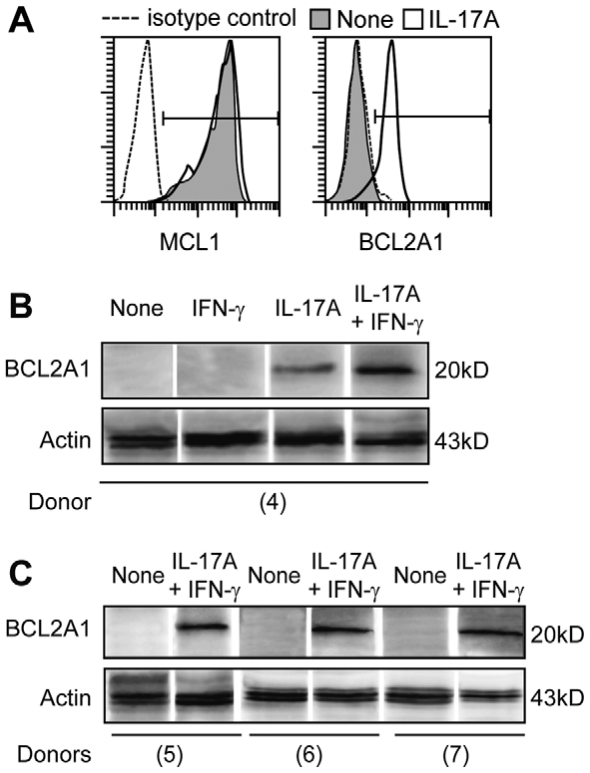
MCL1 and BCL2A1 protein expression in IL-17A-treated DC. (**A**) Intracellular expression of MCL1 and BCL2A1 in DC treated (white) or not (gray) with IL-17A, at day 7, representative of n>3, SD<2%. (**B,C**) Western blot analysis of BCL2A1 versus actin protein expressions in DC cultured with indicated cytokines, lyzed at day 5 for 4 donors (4) to (7), in separated experiments.

We studied the Bcl-2 family gene expression that regulates cell survival in monocyte-derived DC in four donors. Untreated DC expressed five pro-apoptotic genes (*BAX*, *BCL2L11*, *BCL2L13*, *BID* and *BNIP3*) and one pro-survival gene (*MCL1*) (**Fig. 3A**). Untreated DC hardly survived 48 h (**Fig. 1A**), indicating that *MCL1* was not sufficient to maintain DC survival. Large scale transcriptome analysis was performed in parallel on the same DC cultured 12 days with IL-17A, alone or combined with IFN-γ. As generally observed for hematopoietic tissue, we found that IL-17RA was expressed in immature DC, whereas IL-17RC mRNA was below the threshold level of detection (data not shown). Exposure to IL-17A, which induced long-term survival, resulted in a major activation of *BCL2A1* transcription, while *MCL1*, *BCL2L11* and *BID* mRNA amounts decreased to about half, and *BNIP3* increased three-fold (**Fig. 3A**). Contrary to the pro-apoptotic genes whose expression was overall weak, both *MCL1 and BCL2A1* pro-survival genes were highly expressed in IL-17A-treated DC, as illustrated by the fold change heat map, after 12 days of IL-17A treatment (**Fig. 3B**). Early induction of *BCL2A1* by IL-17A was further confirmed by quantitative RT-PCR in three additional donors at day two of culture (**Fig. 3C**). The increase of fold inductions between day 2 and day 12 (**Fig. 3C and 3A**) revealed high accumulation of *BCL2A1* mRNA under the control of IL-17A. We then studied intracellular expression of MCL1 and BCL2A1 proteins by flow cytometry after membrane permeabilization (**Fig. 4A**) or by western blot analysis after cell lysis (**Fig. 4B and 4C**). Both techniques established that IL-17A induced BCL2A1 in MCL1^+^ DC from three additional donors. Despite its role on cell fusion, addition of IFN-γ regulated neither *BCL2A1* mRNA expression (**Fig. 3A-C**), nor BCL2A1 protein expression (**Fig. 4C**). Therefore, IL-17A-treated DC, with or without IFN-γ, strongly co-express the pro-survival proteins MCL1 and BCL2A1.

### BCL2A1 expression induced by IL-17A enhances human DC lifespan

**Figure 5:**
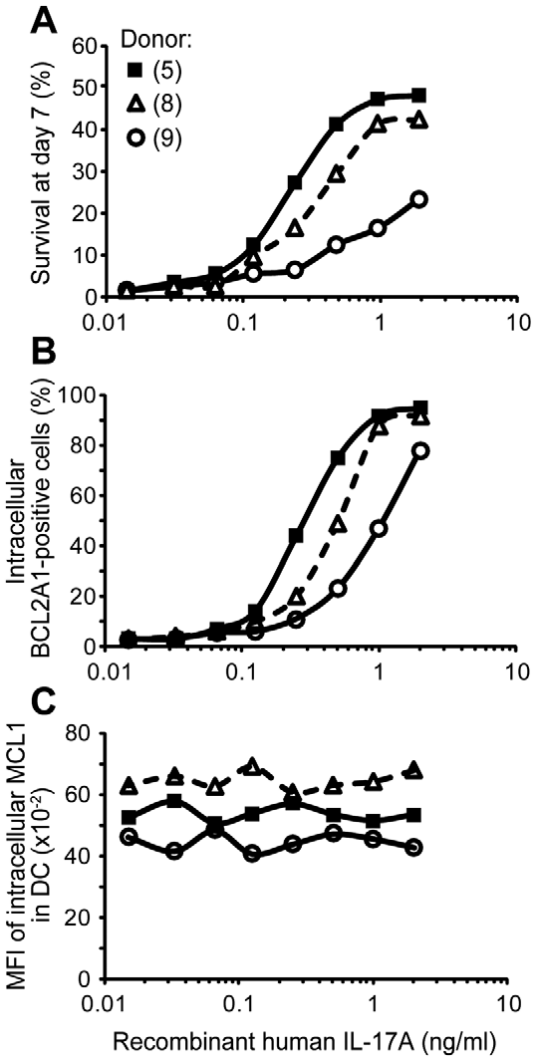
Correlation studies between cell survival, MCL1 and BCL2A1 expression in IL-17A-treated DC. DC were cultured 7 days with IL-17A (eight doses from 0.016 to 2 ng/ml). Flow cytometry analysis of (**A**) survival, assessed by DiOC_6_ and PI staining, (**B**) BCL2A1 and (**C**) MCL1 intracellular expressions for three donors (5, 8, 9), in separated experiments, SD<2%.

Our results suggest that co-expression of the *MCL1* and *BCL2A1* pro-survival genes in DC may support IL-17A-dependent long-term cell survival. Dose responses of IL-17A were performed on DC from three donors and showed that IL-17A increased DC survival in a dose-dependent manner (plateau at 1–2 ng/ml) (**Fig. 5A**). As introduction of sh/siRNA by lipofection or nucleofection affected survival and phenotype of stimulated DC in the control experiment, we could not directly investigate the consequences of *BCL2A1* mRNA blockade (data not shown). Therefore, we used a statistical approach to study the putative link introduced by IL-17A treatment on the three parameters that we quantified: DC survival, and BCL2A1 and MCL1 expression (**Fig. 5A–C**). Statistical analysis showed that the ability of DC to survive was correlated to BCL2A1 expression by a two parameter degree 2 polynomial with a correlation factor >0.978 (data not shown).

In conclusion, although constitutive MCL1 expression is sufficient for short-term (48 h) survival of DC, additional high and persistent BCL2A1 expression, induced by IL-17A, is correlated with a long-term lifespan of DC (beyond 12 days).

### IL-17A induced NF-κB translocation upstream to BCL2A1 expression in human DC

**Figure 6:**
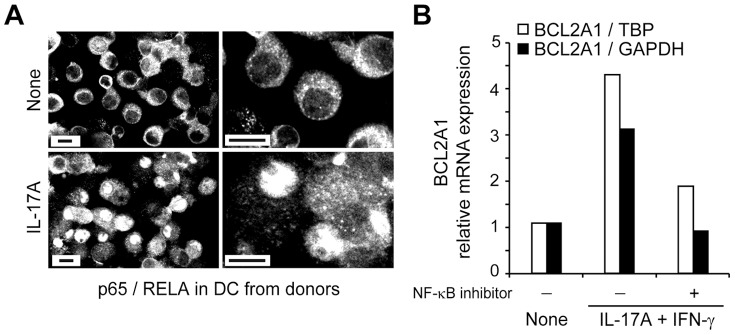
Role of classical NF-κB pathway activation in BCL2A1 induction mediated by IL-17A. (**A**) Nuclear translocation of the NF-κB subunit p65/RelA in DC, after 1 h of IL-17A treatment. Right panels are zoomed views of the left panels, representative of n = 3 donors. Scale bars, 10 µm. (**B**) Effect of the classical NF-κB pathway inhibitor Bay-11-7085 on *BCL2A1* mRNA levels measured by real-time PCR, 48 h after cytokine treatment. The relative gene expression is compared with either TBP or GAPDH.

The nuclear factor p65/RelA protein, a member of the NF-κB transcription factor family, is a known regulator of *BCL2A1* gene expression [17], expressed in immature DC. Therefore, we next investigated p65/RelA translocation in DC nuclei by immunofluorescence detection (**Fig. 6A**). In untreated immature DC, p65/RelA was located in the cytoplasm, as demonstrated by fluorescent cytoplasm and black nuclei analyzed by confocal microscopy. One hour after IL-17A stimulation, fluorescence stained nuclei in about 90% of the DC. Moreover, the NF-κB inhibitor Bay-11-7085 blocked IL-17A-dependent *BCL2A1* mRNA induction in healthy DC (**Fig. 6B**). In conclusion, IL-17A stimulation induces NF-κB p65/RelA subunit translocation, thus activating a high and stable expression of the pro-survival BCL2A1 protein in human DC.

### BCL2A1-expressing DC develop chemoresistance

**Figure 7:**
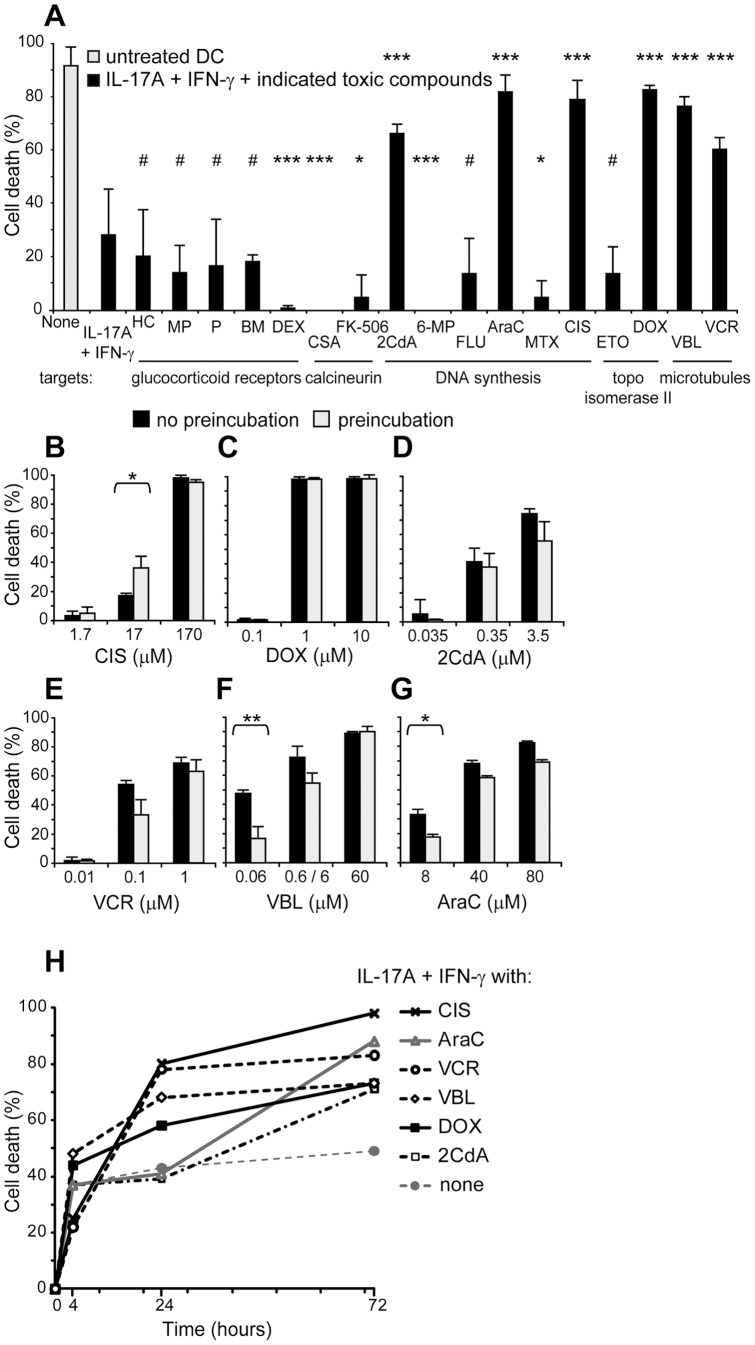
Chemoresistance of IL-17A and IFN-γ-treated DC in the presence of 17 chemotherapy agents. (**A–H**) Flow cytometry analysis of cell death assessed by DiOC_6_ and PI staining. (**A**) DC were cultured 72 h in medium alone (gray) or with IL-17A and IFN-γ (black). Indicated toxic compounds were added together with cytokines and cell death were analyzed 4 h, 24 h or 72 h later. Results of the screening are presented at optimal killing effect (see Methods S1 and Table S1 for full names of toxic compounds and optimal dose). p-values : #, not significant: *, significant p<0.05; **, very significant p<0.01; ***, highly significant p<0.001. For the six toxic compounds (2CdA, AraC, CIS, DOX, VBL and VCR) that killed cytokine-stimulated DC: (**B–G**) Dose response study at optimal time, 24 or 72 h after addition of toxic compounds, according to (H). Toxic compounds were added in DC cultures either concomitantly (black) or 24 h later (gray, preincubation) stimulation with IL-17A and IFN-γ. (**H**) Kinetic study at optimal concentration, according to (B–G). Mean and SD of a triplicate experiment representative of (**A**) n = 5, (**B–G**) n = 3 and (**H**) n = 3, SD below 10%.

BCL2A1 is known to confer chemoresistance in B cell leukemia [18]. Consequently, we investigated whether DC also acquire chemoresistance when they express BCL2A1. *In vivo*, it would be interesting to kill cancer cells, while DC survive to prime IFN-γ-mediated anti-tumor activity. Therefore we studied chemoresistance of IL-17A-stimulated dendritic cells, in the presence of IFN-γ, *in vitro*. We tested 17 chemotherapy agents targeting glucocorticoid receptors, calcineurin, DNA synthesis, topoisomerase II or microtubules (**Fig. 7A** **, Table S1**). DC were cultured with IL-17A and IFN-γ. Toxic compounds were added together with cytokines. We analyzed cell death 4, 24 and 72 hours later. We observed no cytotoxic effect of four glucocorticoids, fludarabine or etoposide, and unexpected pro-survival effects of dexamethasone, two calcineurin inhibitors, 6-mercaptopurine and methotrexate. On the contrary, cladribine (2CdA), cytarabine (AraC), cisplatin (CIS), doxorubicin (DOX), vinblastine (VBL) and vincristine (VCR) killed cytokine-stimulated DC. Noticeably, DC were resistant to some drugs but not to others, even if the drugs belong to the same group. 2CdA, AraC, CIS, DOX, VBL and VCR displayed the same effect on IL-17A-treated DC, with or without IFN-γ (data not shown). Dose response studies showed that CIS, DOX and 2CdA killed only at high doses, exceeding the therapeutic doses (**Fig. 7B–D**) while, interestingly, VCR, VBL and AraC killed at moderate doses (**Fig. 7E–G**). We observed that 24 h of pre-incubation with the cytokines facilitated DC killing by CIS while, it conversely protected DC from death induced by low doses of VBL or AraC. VBL and DOX were effective already at four hours (**Fig. 7H**). Optimal death was obtained at 24 hours with VCR and CIS, and at 72 hours with AraC and 2CdA. Altogether, these data demonstrate that IL-17A and IFN-γ-stimulated DC are chemoresistant to 11 of the 17 chemotherapy agents tested but they are highly sensitive to VBL and AraC, at concentrations observed in clinical settings.

### VBL and AraC induced cell death by targeting MCL1 but not BCL2A1

**Figure 8:**
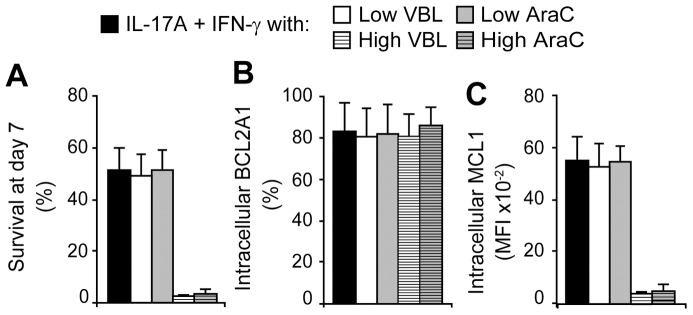
Survival and phenotype of DC cultured with VBL or AraC. DC were stimulated with IL-17A and IFN-γ for 7 days. According to their toxicity kinetic, VBL or AraC were added in the culture at day 6 or 5, respectively. (**A**) Cell survival assessed by DiOC_6_ and PI staining, at day 7. (**B**) BCL2A1 and (**C**) MCL1 intracellular expressions were measured prior DiOC_6_ and PI staining. “Low” doses of VBL and AraC were 0.06 and 4 µM, respectively. “High” doses were ten times more. Mean and SD of n = 5.

40–60% of MCL1^+^BCL2A1^+^ DC survived in the presence of low doses of VBL or AraC while high doses killed IL-17A and IFN-γ-treated DC (**Fig. 8A**). We next investigated the role of VBL and AraC on MCL1 and BCL2A1 expression in DC. When DC underwent cell death in the presence of high doses of VBL or AraC, BCL2A1 expression did not change contrary to MCL1 expression which was strongly decreased by either VBL or AraC (**Fig. 8B and 8C**). We stained microtubules, actin and nuclei to visualize that cytokines induced a giant microtubule network in giant cells (**Fig. S2**) which is disorganized by VBL. In addition, it was possible to reverse BCL2A1 expression by adding anti-IL-17A blocking antibodies after BCL2A1 induction by IL-17A and this strategy ten times increased cell sensitivity to either VBL or AraC (data not shown).

Therefore, we demonstrated that while survival is prolonged by IL-17A-mediated BCL2A1 up-regulation, VBL and AraC induces death by down-regulating MCL1 expression, without affecting BCL2A1. Overall, in the absence of MCL1 expression, BCL2A1 alone is unable to maintain DC alive, indicating that both BCL2A1 and MCL1 are mandatory to establish long-term DC survival.

## Discussion

Tumor-associated DC can either mount an anti-tumoral immune response or support tumor tolerance, thus their biology is central in carcinogenesis. Here, we provide original evidence that the usual pattern of short-time (two days) DC lifespan is significantly extended beyond 12 days by exposure of DC to IL-17A, *in vitro*. Interestingly, the pro-inflammatory IL-17A up-regulates macrophage markers in DC and induces, via NF-κB, the expression of BCL2A1. The long-term surviving myeloid cells, expressing both BCL2A1 and MCL1, do not proliferate but develop a chemoresistance to 11 of the 17 chemotoxic agents tested. However, these chemoresistant myeloid cells were highly sensitive to VBL and AraC that target MCL1. These data provide a rationale for novel therapeutic approaches, targeting both MCL1 and BCL2A1 in myeloid cells, which may be useful in the treatment of cancer whose development is sustained by tolerogenic DC.

When IL-17A interacts with its receptor chain IL-17RA, Act1 and TRAF6 are recruited and further activate NF-κB [19]. Among the five NF-κB proteins known in mammals, only RelA was expressed in the transcriptome of IL-17A-stimulated DC. Furthermore, a RelA responsive element is located in the promoter of *BCL2A1* gene and positively regulates BCL2A1 expression [17]. In DC, we demonstrate that IL-17A activates NF-κB p65/RelA subunit translocation, which then induces BCL2A1 transcription, as illustrated by the shut off operated by the NF-κB inhibitor Bay-11-7085. Independently of BCL2A1, it was previously demonstrated that balance between NF-κB and JNK/AP-1 activity controls DC apoptosis: JNK/AP-1 activity is under negative feedback control of NF-κB and can execute apoptosis in DC [20]. Therefore NF-κB inhibitors induce DC death, by licensing JNK/AP-1-mediated apoptosis. Finally, downstream of IL-17A signaling, nuclear translocation of NF-κB provides the basis both for inhibiting JNK/AP-1-dependent apoptosis, and for up-regulating BCL2A1-dependent survival, thus indicating an important unexpected function of BCL2A1 in DC, during IL-17A-driven inflammation.

Our data demonstrate that IL-17A-induced BCL2A1 expression was correlated to the acquisition of long-term survival. The function of BCL2A1 is to counteract the activation of the BH3-only proteins BAK and BAX. We showed that DC highly express mRNA of BCL2L11 and BID. BH3 profiling and fluorescence polarization assay has indicated that BCL2A1 binds BCL2L11, BID and BBC3/PUMA [21]. Therefore, inhibition of the intrinsic apoptosis by BCL2A1 that ensures long-term survival of IL-17A-treated DC may result from the sequestration of BCL2L11 and BID BH3-proteins by BCL2A1.

In humans, BCL2A1 is widely expressed in lung, small intestine, testis and smooth muscle [22]. Early involved in fetal liver hematopoiesis, it is also induced later in life, upon TNF-α and IL-1β treatment, in activated endothelial cells which then become protected from death [23]. It has been suggested that BCL2A1 expression protects endothelial cells from death when they are exposed to inflammation-associated cellular toxins. We showed that IL-17A is a pro-inflammatory cytokine that induces BCL2A1 expression in human DC, protecting them from death. Due to the specific function of DC in activating naïve T cells, we suggest that, in addition to endothelial cell protection, IL-17A could be a molecular key to support the development of a chronic inflammation mediated by multiple DC-T cell crosstalks, due to increased life of MCL1^+^BCL2A1^+^ DC.

High BCL2A1 expression has been found to correlate with more severe cases of progressive chronic lymphocytic leukemia, indicating that BCL2A1 contributes to apoptosis resistance [24], [25]. However, transgenic BCL2A1 mice do not develop lymphomas [26], [27], suggesting that either BCL2A1 overexpression is not sufficient for tumorigenesis or BCL2A1 functions differ between mice and humans, as is the case of BCL2A1 cellular distribution. More recently, human BCL2A1 mRNA was found to be overexpressed in various solid tumors such as stomach, colon cancer, and breast cancer, skin squamous cell carcinoma, hepatocellular carcinoma and melanoma [28]. The highest expression is predominantly associated with advanced disease stages [29]. Thus, human BCL2A1 is overexpressed in a variety of cancer cells, including hematological malignancies and solid tumors and contribute to tumor progression. In addition to BCL2A1 expression, we also identified a pre-M2 phenotype in IL-17A-treated DC, opening the view that, in the absence of IFN-γ, IL-17A-dependent myeloid cell plasticity may give rise to M2-like tumor-associated macrophages [30]. They generally present an IL-12^low^IL-10^high^ phenotype, less antigen presentation and tumoricidal capacity, and high expression of angiogenic factors such as VEGF. We found that IL-17A-treated DC highly express CCL22. In breast tumors, this chemokine attracts peripheral blood CCR4^+^ regulatory T cells, which are then selectively activated in lymphoid tumor infiltrates, thus preventing effector T cell activation, while sustaining immune escape, and ultimately tumor progression [31].

High expression of BCL2A1 is associated to chemoresistance against fludarabine or etoposide in progressive chronic lymphocytic leukemia cells, *in vitro* [18], [32]. Conversely, silencing BCL2A1 sensitizes T or B cancer cells to apoptosis induced by chemotherapy or anti-CD19 biotherapy, in both cell lines and cells from patients [33]. We document that IL-17A-treated DC develop chemoresistance to a wide range of toxic compounds (11/17) when they acquire BCL2A1 expression. It was surprising that DC were resistant to some drugs and not others, even when they belong to the same group. However, we could find some explanations in the literature. Cellular uptake of fludarabine requires dephosphorylation by phosphatases, in the body circulation, to produce arabinosyl-2-fluoroadenine, which can then be transported into the cells and become active [34]; therefore failure of fludarabine to kill IL-17A-treated DC, *in vitro*, may be due to its inability to enter into DC. Contrary to etoposide that affects only dividing cells, doxorubicin could affect non-dividing cells and the difference sensitivities to these anthracyclin antibiotics is already known for freshly generated monocyte-derived DC [35]. It may come either from the higher ability of doxorubicin to produce superoxide anions that induce apoptosis, or from a differential drug effect on DNA integrity, as previously shown on cancer cell lines [36]. Indeed, reversal of DNA lesions rather than the occurrence of DNA breaks plays a major role in cell survival. DNA lesions produced by doxorubicin persisted and even increased following drug removal while reversal of etoposide-induced DNA breaks was associated to cell survival. IL-17A-treated DC are nevertheless sensitive to high doses of VBL and AraC, which target only MCL1. VBL appears to be more potent than AraC, maybe due to its additional activity on microtubules. Indeed, both VBL and VCR, two related vinca alkaloids with close formulas, were previously known as microtubule depolymerizing drugs [37]. Interestingly, our studies extend the recently documented MCL1 degradation after VCR treatment [38] also to therapy with VBL.

Anti-IL-17A biotherapy is currently developed for the treatment of inflammatory diseases. Transcriptional profiling recently characterized the tumor microenvironment and host inflammatory response in diffuse large B-cell lymphoma, which expresses BCL2A1 [14]. According to the data set analysis, the authors suggested that tumor inflammation was chronic but ineffective. They proposed to identify tumors with pre-existing abundant T cell and DC infiltrates and to further characterize their associated underlying immune response to perform immunomodulatory approaches as a treatment. When the DC are tolerogenic, it is important to kill them as well as tumor cells, by suitable therapy. IL-17A may stimulate angiogenesis and long-term survival of tolerogenic DC, thus driving tumor growth. Myeloid cells mediate suppression either directly or indirectly by activating regulatory T cells. Concomitant expressions of IL-17A, with or without IFN-γ, pro-angiogenic VEGF, pro-survival BCL2A1 and suppressive IL-10 [39] or free radical peroxynitrite [40] may indicate that IL-17A strongly supports cancer development. Our data obtained with human primary DC cultures delineate future personalized medicine combining anti-IL-17A biotherapy with (VBL- or AraC-based) chemotherapy to counteract cancer cell survival in an IL-17A-rich tolerogenic microenvironment.

## Supporting Information

Figure S1 **CSF ligand and receptor mRNA expression in monocytes, DC and IL-17A-treated DC.** mRNA intensities (microarray) of CD68, CD14, colony stimulating factor ligands and genes in freshly purified monocytes, DC at day 0 and after 12 days of culture with IL-17A, representative of n = 4.
(PDF)

Figure S2 **Cytoskeleton study of DC stimulated with IL-17A and IFN-γ and then treated with VBL.** Confocal microscopy pictures after triple immunofluorescence staining of IL-17A and IFN-γ-stimulated DC, fixed at day 12, two hours after addition of (c,d) VBL or (a,b) none. Hoechst blue staining localized nuclei while green phalloidin revealed actin cytoskelon. Red color stained either (a,c) tubulin or (b,d) vinculin. Arrow-heads indicate (a) double tubulin-actin staining in yellow or (d) bright blue fragmented DNA inside nuclei of apoptotic cells. Stars localize giant cells. Arrow indicates keel-like structures of variable size. Enlarged inset picture shows a ten-time magnification inside the podosome region. Scale bars, 50 µm (5×10 µm), representative of n = 3.
(PDF)

Table S1 Class, name, in vitro range and optimal doses to kill IL-17A and IFN-γ-stimulated DC, in vivo approximate clinical doses of chemotherapy agents.
(PDF)

Methods S1 Supplementary methods.
(PDF)

## References

[1] Coussens LM, Werb Z (2002) Inflammation and cancer. Nature 420: 860–867. 12490959 10.1038/nature01322 PMC2803035

[2] Sarra M, Pallone F, Macdonald TT, Monteleone G (2010) IL-23/IL-17 axis in IBD. Inflamm Bowel Dis 16: 1808–1813. 20222127 10.1002/ibd.21248

[3] Katsifis GE, Rekka S, Moutsopoulos NM, Pillemer S, Wahl SM (2009) Systemic and local interleukin-17 and linked cytokines associated with Sjogren's syndrome immunopathogenesis. Am J Pathol 175: 1167–1177. 19700754 10.2353/ajpath.2009.090319 PMC2731135

[4] Korn T, Bettelli E, Oukka M, Kuchroo VK (2009) IL-17 and Th17 Cells. Annu Rev Immunol 27: 485–517. 19132915 10.1146/annurev.immunol.021908.132710

[5] Gaffen SL (2009) The role of interleukin-17 in the pathogenesis of rheumatoid arthritis. Curr Rheumatol Rep 11: 365–370. 19772832 10.1007/s11926-009-0052-y PMC2811488

[6] Ely LK, Fischer S, Garcia KC (2009) Structural basis of receptor sharing by interleukin 17 cytokines. Nat Immunol 10: 1245–1251. 19838198 10.1038/ni.1813 PMC2783927

[7] Numasaki M, Fukushi J, Ono M, Narula SK, Zavodny PJ, et al (2003) Interleukin-17 promotes angiogenesis and tumor growth. Blood 101: 2620–2627. 12411307 10.1182/blood-2002-05-1461

[8] Benchetrit F, Ciree A, Vives V, Warnier G, Gey A, et al (2002) Interleukin-17 inhibits tumor cell growth by means of a T-cell-dependent mechanism. Blood 99: 2114–2121. 11877287 10.1182/blood.v99.6.2114

[9] Cheong C, Matos I, Choi JH, Dandamudi DB, Shrestha E, et al (2010) Microbial stimulation fully differentiates monocytes to DC-SIGN/CD209(+) dendritic cells for immune T cell areas. Cell 143: 416–429. 21029863 10.1016/j.cell.2010.09.039 PMC3150728

[10] Coury F, Annels N, Rivollier A, Olsson S, Santoro A, et al (2008) Langerhans cell histiocytosis reveals a new IL-17A-dependent pathway of dendritic cell fusion. Nat Med 14: 81–87. 18157139 10.1038/nm1694

[11] Frenzel A, Grespi F, Chmelewskij W, Villunger A (2009) Bcl2 family proteins in carcinogenesis and the treatment of cancer. Apoptosis 14: 584–596. 19156528 10.1007/s10495-008-0300-z PMC3272401

[12] Kozopas KM, Yang T, Buchan HL, Zhou P, Craig RW (1993) MCL1, a gene expressed in programmed myeloid cell differentiation, has sequence similarity to BCL2. Proc Natl Acad Sci U S A 90: 3516–3520. 7682708 10.1073/pnas.90.8.3516 PMC46331

[13] Feuerhake F, Kutok JL, Monti S, Chen W, LaCasce AS, et al (2005) NFkappaB activity, function, and target-gene signatures in primary mediastinal large B-cell lymphoma and diffuse large B-cell lymphoma subtypes. Blood 106: 1392–1399. 15870177 10.1182/blood-2004-12-4901

[14] Monti S, Savage KJ, Kutok JL, Feuerhake F, Kurtin P, et al (2005) Molecular profiling of diffuse large B-cell lymphoma identifies robust subtypes including one characterized by host inflammatory response. Blood 105: 1851–1861. 15550490 10.1182/blood-2004-07-2947

[15] Oltersdorf T, Elmore SW, Shoemaker AR, Armstrong RC, Augeri DJ, et al (2005) An inhibitor of Bcl-2 family proteins induces regression of solid tumours. Nature 435: 677–681. 15902208 10.1038/nature03579

[16] Rivollier A, Mazzorana M, Tebib J, Piperno M, Aitsiselmi T, et al (2004) Immature dendritic cell transdifferentiation into osteoclasts: a novel pathway sustained by the rheumatoid arthritis microenvironment. Blood 104: 4029–4037. 15308576 10.1182/blood-2004-01-0041

[17] D'Souza BN, Edelstein LC, Pegman PM, Smith SM, Loughran ST, et al (2004) Nuclear factor kappa B-dependent activation of the antiapoptotic bfl-1 gene by the Epstein-Barr virus latent membrane protein 1 and activated CD40 receptor. J Virol 78: 1800–1816. 14747545 10.1128/JVI.78.4.1800-1816.2004 PMC369510

[18] Morales AA, Olsson A, Celsing F, Osterborg A, Jondal M, et al (2005) High expression of bfl-1 contributes to the apoptosis resistant phenotype in B-cell chronic lymphocytic leukemia. Int J Cancer 113: 730–737. 15499630 10.1002/ijc.20614

[19] Chang SH, Dong C (2011) Signaling of interleukin-17 family cytokines in immunity and inflammation. Cell Signal 23: 1069–1075. 21130872 10.1016/j.cellsig.2010.11.022 PMC3078175

[20] Kriehuber E, Bauer W, Charbonnier AS, Winter D, Amatschek S, et al (2005) Balance between NF-kappaB and JNK/AP-1 activity controls dendritic cell life and death. Blood 106: 175–183. 15755895 10.1182/blood-2004-08-3072

[21] Certo M, Del Gaizo Moore V, Nishino M, Wei G, Korsmeyer S, et al (2006) Mitochondria primed by death signals determine cellular addiction to antiapoptotic BCL-2 family members. Cancer Cell 9: 351–365. 16697956 10.1016/j.ccr.2006.03.027

[22] Karsan A, Yee E, Kaushansky K, Harlan JM (1996) Cloning of human Bcl-2 homologue: inflammatory cytokines induce human A1 in cultured endothelial cells. Blood 87: 3089–3096. 8605321

[23] Karsan A, Yee E, Harlan JM (1996) Endothelial cell death induced by tumor necrosis factor-alpha is inhibited by the Bcl-2 family member, A1. J Biol Chem 271: 27201–27204. 8910286 10.1074/jbc.271.44.27201

[24] Macaire H, Riquet A, Moncollin V, Biemont-Trescol MC, Duc Dodon M, et al (2012) Tax Protein-induced Expression of Antiapoptotic Bfl-1 Protein Contributes to Survival of Human T-cell Leukemia Virus Type 1 (HTLV-1)-infected T-cells. J Biol Chem 287: 21357–21370. 22553204 10.1074/jbc.M112.340992 PMC3375557

[25] Olsson A, Norberg M, Okvist A, Derkow K, Choudhury A, et al (2007) Upregulation of bfl-1 is a potential mechanism of chemoresistance in B-cell chronic lymphocytic leukaemia. Br J Cancer 97: 769–777. 17726463 10.1038/sj.bjc.6603951 PMC2360383

[26] Chuang PI, Morefield S, Liu CY, Chen S, Harlan JM, et al (2002) Perturbation of B-cell development in mice overexpressing the Bcl-2 homolog A1. Blood 99: 3350–3359. 11964303 10.1182/blood.v99.9.3350

[27] McDonnell TJ, Korsmeyer SJ (1991) Progression from lymphoid hyperplasia to high-grade malignant lymphoma in mice transgenic for the t(14; 18). Nature 349: 254–256. 1987477 10.1038/349254a0

[28] Vogler M (2012) BCL2A1: the underdog in the BCL2 family. Cell Death Differ 19: 67–74. 22075983 10.1038/cdd.2011.158 PMC3252829

[29] Riker AI, Enkemann SA, Fodstad O, Liu S, Ren S, et al (2008) The gene expression profiles of primary and metastatic melanoma yields a transition point of tumor progression and metastasis. BMC Med Genomics 1: 13. 18442402 10.1186/1755-8794-1-13 PMC2408576

[30] Biswas SK, Mantovani A (2010) Macrophage plasticity and interaction with lymphocyte subsets: cancer as a paradigm. Nat Immunol 11: 889–896. 20856220 10.1038/ni.1937

[31] Gobert M, Treilleux I, Bendriss-Vermare N, Bachelot T, Goddard-Leon S, et al (2009) Regulatory T cells recruited through CCL22/CCR4 are selectively activated in lymphoid infiltrates surrounding primary breast tumors and lead to an adverse clinical outcome. Cancer Res 69: 2000–2009. 19244125 10.1158/0008-5472.CAN-08-2360

[32] Wang CY, Guttridge DC, Mayo MW, Baldwin AS Jr (1999) NF-kappaB induces expression of the Bcl-2 homologue A1/Bfl-1 to preferentially suppress chemotherapy-induced apoptosis. Mol Cell Biol 19: 5923–5929. 10454539 10.1128/mcb.19.9.5923 PMC84448

[33] Brien G, Trescol-Biemont MC, Bonnefoy-Berard N (2007) Downregulation of Bfl-1 protein expression sensitizes malignant B cells to apoptosis. Oncogene 26: 5828–5832. 17353899 10.1038/sj.onc.1210363

[34] Johnson SA (2000) Clinical pharmacokinetics of nucleoside analogues: focus on haematological malignancies. Clin Pharmacokinet 39: 5–26. 10926348 10.2165/00003088-200039010-00002

[35] Chao D, Bahl P, Houlbrook S, Hoy L, Harris A, et al (1999) Human cultured dendritic cells show differential sensitivity to chemotherapy agents as assessed by the MTS assay. Br J Cancer 81: 1280–1284. 10604723 10.1038/sj.bjc.6694366 PMC2362963

[36] Binaschi M, Capranico G, De Isabella P, Mariani M, Supino R, et al (1990) Comparison of DNA cleavage induced by etoposide and doxorubicin in two human small-cell lung cancer lines with different sensitivities to topoisomerase II inhibitors. Int J Cancer 45: 347–352. 2154411 10.1002/ijc.2910450223

[37] Yang H, Ganguly A, Cabral F (2010) Inhibition of cell migration and cell division correlates with distinct effects of microtubule inhibiting drugs. J Biol Chem 285: 32242–32250. 20696757 10.1074/jbc.M110.160820 PMC2952225

[38] Wertz IE, Kusam S, Lam C, Okamoto T, Sandoval W, et al (2011) Sensitivity to antitubulin chemotherapeutics is regulated by MCL1 and FBW7. Nature 471: 110–114. 21368834 10.1038/nature09779

[39] Tanikawa T, Wilke CM, Kryczek I, Chen GY, Kao J, et al (2012) Interleukin-10 ablation promotes tumor development, growth, and metastasis. Cancer Res 72: 420–429. 22123924 10.1158/0008-5472.CAN-10-4627 PMC3261323

[40] Lu T, Ramakrishnan R, Altiok S, Youn JI, Cheng P, et al (2011) Tumor-infiltrating myeloid cells induce tumor cell resistance to cytotoxic T cells in mice. J Clin Invest 121: 4015–4029. 21911941 10.1172/JCI45862 PMC3195459

