# Supplementary material for: Chemoresistance of Human Monocyte-Derived Dendritic Cells Is Regulated by IL-17A
Source: PLoS One. 2013 Feb 18;8(2):e56865. doi: 10.1371/journal.pone.0056865 (PMC3575476; doi:10.1371/journal.pone.0056865)
Supplement: Figure S1 — CSF ligand and receptor mRNA expression in monocytes, DC and IL-17A-treated DC. mRNA intensities (microarray) of CD68, CD14, colony stimulating factor ligands and genes in freshly purified monocytes, DC at day 0 and after 12 days of culture with IL-17A, representative of n = 4. (PDF) [file pone.0056865.s001.pdf]

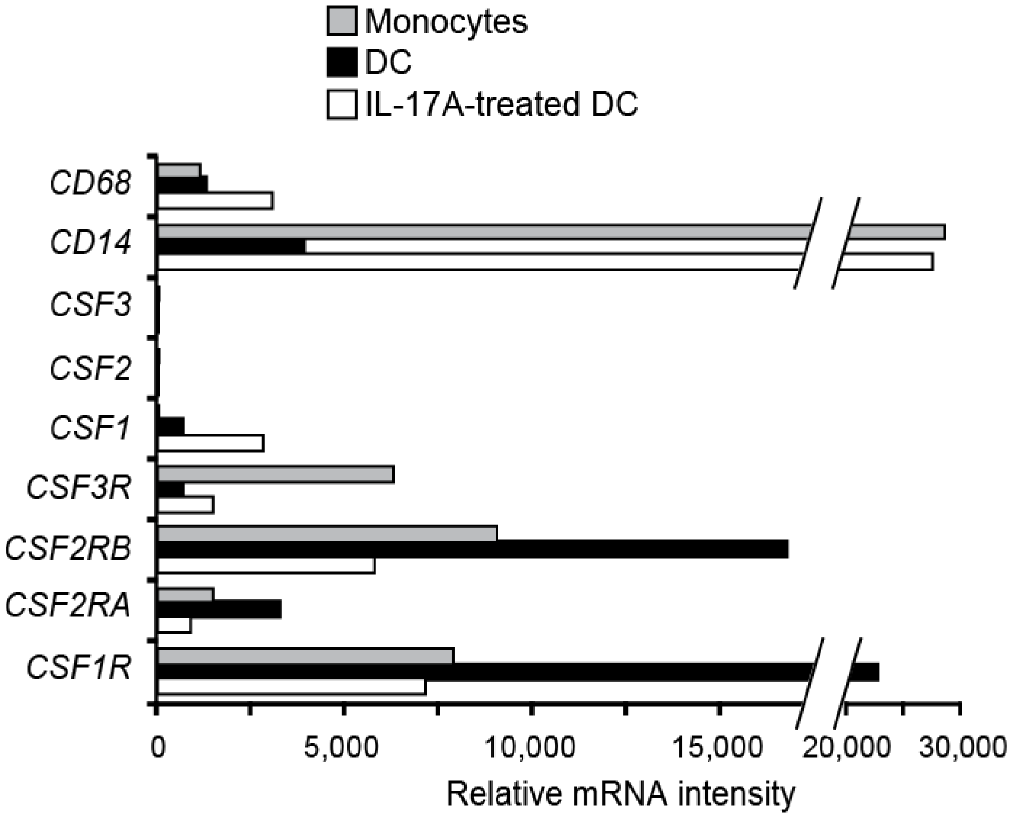

Figure S1 Result – IL-17A-treated DC express CSF1 and CSF1R

Monocytes expressed no colony stimulating factors (*CSF*) mRNAs, while IL-17A highly increased *CSF1* (M-CSF) mRNA synthesis in DC. All CSF receptors were expressed by monocytes. *CSF1R* and both *CSF2R* chain mRNAs (GM-CSF receptor) were up-regulated during their differentiation in DC, while *CSF3R* (G-CSF receptor) was down-regulated. IL-17A-treated DC synthesized *CSF1* and *CSFR1* mRNAs, while *CSF2RA* and *CSF3R* mRNA intensities were very low and down-regulated compare to monocytes. This CSF ligand and receptor pattern of expression showed that monocytes express all CSF receptors, but none of their ligands. According to their CSF receptor expressions, monocyte-derived DC usually respond to both CSF1 and CSF2, however IL-17A polarizes the DC in the CSF1 / CSF1R axis by the up-regulation of CSF1.
