# Supplementary material for: Chemoresistance of Human Monocyte-Derived Dendritic Cells Is Regulated by IL-17A
Source: PLoS One. 2013 Feb 18;8(2):e56865. doi: 10.1371/journal.pone.0056865 (PMC3575476; doi:10.1371/journal.pone.0056865)
Supplement: Figure S2 — Cytoskeleton study of DC stimulated with IL-17A and IFN-γ and then treated with VBL. Confocal microscopy pictures after triple immunofluorescence staining of IL-17A and IFN-γ-stimulated DC, fixed at day 12, two hours after addition of (c,d) VBL or (a,b) none. Hoechst blue staining localized nuclei while green phalloidin revealed actin cytoskelon. Red color stained either (a,c) tubulin or (b,d) vinculin. Arrow-heads indicate (a) double tubulin-actin staining in yellow or (d) bright blue fragmented DNA inside nuclei of apoptotic cells. Stars localize giant cells. Arrow indicates keel-like structures of variable size. Enlarged inset picture shows a ten-time magnification inside the podosome region. Scale bars, 50 µm (5×10 µm), representative of n = 3. (PDF) [file pone.0056865.s002.pdf]

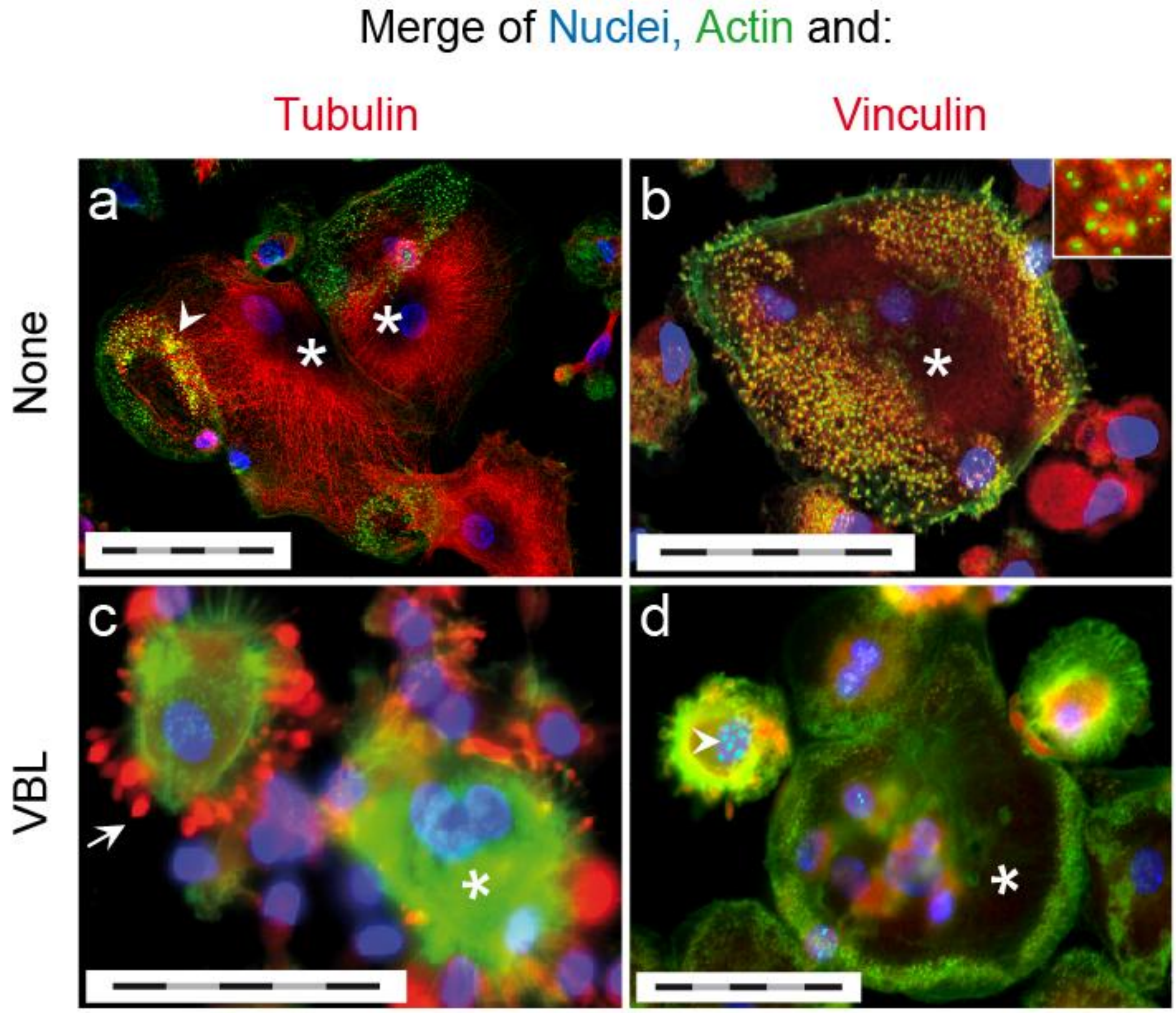

Figure S2 Result – VBL induces microtubules depolymerisation in IL-17A-induced DC-derived giant cells

We stained microtubules, actin and nuclei to visualize that cytokines induced a giant microtubule network in giant cells (a) and observed that VBL disorganized the microtubule network (c). Numerous microtubules end in the cell periphery where actin spots are located, and in some cases, yellow color indicates that they directly contact each other (a, arrow-head). Numerous keel-like unpolymerized tubulin-rich structures were budding from the cytoplasm (c, arrow). Podosomes, identified as spot-like actin-rich structures surrounded by a ring of vinculin, formed at the ventral surface of giant cells (b, inset). Lack of podosomes after VBL treatment, illustrates the requirement of an intact microtubule system for podosome assembly (d). Two hours after VBL addition, we identified the first apoptotic nuclei by condensed spots of DNA (d, arrow-head).
