# Supplementary material for: Chemoresistance of Human Monocyte-Derived Dendritic Cells Is Regulated by IL-17A
Source: PLoS One. 2013 Feb 18;8(2):e56865. doi: 10.1371/journal.pone.0056865 (PMC3575476; doi:10.1371/journal.pone.0056865)
Supplement: Table S1 — Class, name, in vitro range and optimal doses to kill IL-17A and IFN-γ-stimulated DC, in vivo approximate clinical doses of chemotherapy agents. (PDF) [file pone.0056865.s003.pdf]

Table S1: *Class, name, in vitro range and optimal doses to kill IL-17A and IFN-γ-stimulated DC, in vivo approximate clinical doses of chemotherapy agents*

**Class**	**Abbreviations: name**	***In vitro* range, optimal dose for killing (μM)**	**Clinical dose (μM)**	**Targets**
Glucocorticoid	HC : Hydrocortisone	0.01-100, no killing	10	Immune system
Glucocorticoid	MP: Methylprednisolone	0.01-100, no killing	50	Immune system
Glucocorticoid	P: Prednisolone	0.01-100, no killing	5	Immune system
Glucocorticoid	BM: Betamethasone	0.01-100, no killing	1	Immune system
Glucocorticoid	DEX: Dexamethasone	0.01-100, no killing	0.5	Immune system
11 aminoacid cyclic peptide	CSA: Cyclosporine A	0.008-80, no killing	0.5	Immune system, calcineurin
Macrolide	FK-506:Tacrolimus	0.0001-1, no killing	0.02	Immune system, calcineurin
Purine analogue	2CdA : Cladribine	0.00035-3.5, 3	0.02	DNA synthesis
Purine analogue	6-MP: 6-mercaptopurine	0.05-500, no killing	5	DNA synthesis
Purine analogue	FLU: Fludarabine	0.01-10, no killing	5	DNA synthesis
Pyrimidine analogue	AraC : Cytarabine	0.8-800, 40	14-140	DNA synthesis, *MCL1*
Folate acid antagonist	MTX: Methotrexate	5-5,000, no killing	1.5-75	DNA synthesis
Organometallic complex, purine linker	CIS: Cisplatin	0.17-170, 100	20	DNA synthesis
Alkaloid	ETO:Etoposide	0.1-100, no killing	12	Topoisomerase II
Anthracycline antibiotic intercalating agent	DOX: Doxorubicin	0.001-10, 1	0.2	Topoisomerase II
Alkaloid	VBL: Vinblastine	0.06-60, 0.6	1.5	Microtubule function
Alkaloid	VCR:Vincristine	0.0001-1, 1	0.2	Microtubule function
