# Supplementary material for: Chemoresistance of Human Monocyte-Derived Dendritic Cells Is Regulated by IL-17A
Source: PLoS One. 2013 Feb 18;8(2):e56865. doi: 10.1371/journal.pone.0056865 (PMC3575476; doi:10.1371/journal.pone.0056865)
Supplement: Methods S1 — Supplementary methods. (PDF) [file pone.0056865.s004.pdf]

Methods S1

Chemotherapeutic compounds and calculation of physiological doses

The magnitude of the microenvironment concentration around cells, *in vivo*, following clinical dose administration, was calculated by approximating that the drug could be distributed in half of the body aqueous volume (30L) with the formula: [(injected concentration) x injected volume] / 30. The results are in the range of those indicated by pharmacokinetics studies. The following table includes class, target: name (abbreviation), *in vitro* range (optimal dose to kill IL-17A and IFN-γ-stimulated DC when efficient) in µM, and clinical dose in µM, respectively.

Affymetrix Genechip study, microarray analysis:

Target labeling: Microarray analysis was performed using a high-density oligonucleotide array (Genechip human genome U133 Plus 2.0, Affymetrix, Santa Clara, CA, USA). Labeled target for microarray hybridization was prepared using the Genechip expression 3' Amplification One-cycle target labeling (Affymetrix). Briefly, total RNA (2 microg) was converted into double stranded cDNA with a modified oligo(dT)24-T7 promoter primer. After purification, cDNA was converted into cRNA and biotinylated using the IVT labeling kit (Affymetrix). Reaction was carried out for 16 hours at 37°C then at the end of incubation biotin-labeled cRNA was purified by the Genechip sample clean up module (Affymetrix). cRNA quantification was performed with a nanodrop and quality checked with the bioanalyzer 2100 (Agilent technologies, Inc, Palto Alto, CA, USA).

Arrays hybridization and scanning: Hybridization was performed following Affymetrix protocol (http://www.affymetrix.com). Briefly, 20 microg of labeled cRNA was fragmented, mixed in hybridization buffer (50 pM control oligo B2, 1X eukaryotic hybridization controls, 0,1mg/ml Herring sperm DNA, 0.5 mg/ml BSA and 1x hybridization buffer, 10% DMSO for a total volume of 300 ul), denaturated during 5 minutes at 95°C and hybridized on chip during 16 hours at 45°C with constant mixing by rotation at 60 rpm in an Genechip hybridization oven 640 (Affymetrix). After hybridization, arrays were washed and stained with streptavidin-phycoerythrin (Invitrogen Corporation, CA, USA) in a fluidic 450 (Affymetrix) according to the manufacturer's instruction. The arrays were read with a confocal laser (Genechip scanner 3000, Affymetrix) and analyzed with GCOS software. Absolute expression transcript levels were normalized for each chip by globally scaling all probe sets to a target signal intensity of 500. The detection metric (presence, absence or marginal) for a particular gene was determined by means of default parameters in the GCOS v

1.4 software (Affymetrix). Quality of RNA amplification and labeling were checked by using *B.subtilis* polyadenylated RNA spikes-in controls (Lys, phe, thr, dap) mixed to RNA sample before performing reverse transcription. Hybridization quality was checked by using *E.coli* biotinylated target (Bio B, BioC, BioD and CRE). Filtering of results was performed using Genespring ver 7.0 software (Agilent technologies Inc, Palto Alto, CA, USA). Heat map was creating using Java TreeView version 1.1.6r2.

Immunocytofluorescence labeling

Cytoskeleton staining: All fixation and labeling procedures were conducted at room temperature. Cells treated or not with VBL were fixed 15 minutes in Busson fixation solution at pH 6.9 (4% paraformaldehyde, 60 mM PIPES, 25 mM HEPES, 20 mM EGTA, 2 mM magnesium acetate, 0.05% glutaraldehyde w/v).3 They were then permeabilized with 0.2% (v/v) TritonX-100 in PBS for 7min. After preincubation 20 minutes in 10% normal human serum in PBS, cells were incubated for 1 hour, with either anti-vinculin (clone hVIN-1 mouse mAb, purified IgG, Sigma) or anti-tubulin (clone TUB.2.1 mouse mAb, Sigma) both diluted 1:100 in 1% BSA 3% normal human serum in PBS. Mouse IgG were used as control. Cells were then labeled with cyanin-5-conjugated goat anti-mouse secondary antibody (Jackson ImmunoResearch Laboratories), diluted 1:200 in the same buffer. At the same time F-actin distribution was revealed after incubation of the cells with Alexa 488-conjugated phalloidin (Invitrogen) according to the manufacturer’s procedure. Finally, DNA nuclei were stained by incubating the cells with 10 mg/ml Hoechst 33342 (Sigma). Cells were extensively washed in PBS and coverslips were mounted in Prolong mounting medium containing anti bleaching agent (Invitrogen). Cells were observed either under a Leica TCS-SP5 laser scanning confocal microscope (Leica, Wetzlar, Germany) using a HCX PL APO 63x 1.4-0.6 oil DIC wd 0.1 mm objective or under an AxioImager Z1 (Zeiss, Jena, Germany) using a 40x-0.75 DIC wd 0.5 mm Plan Neofluar objective. Image acquisition has been done using MetaMorph 7.0 Software (Molecular Devices). To prevent cross-contamination between fluorochromes, each channel was imaged sequentially using the multi-track recording module before merging. Confocal section analyzed do not necessarily include all nuclei thus MGC are defined either by observation of strictly more than two nuclei observed on the section, or by the diameter of the cells above 25µm.
